# The physical and physiological effects of vacuum massage on the different skin layers: a current status of the literature

**DOI:** 10.1186/s41038-016-0053-9

**Published:** 2016-09-19

**Authors:** Peter Moortgat, Mieke Anthonissen, Jill Meirte, Ulrike Van Daele, Koen Maertens

**Affiliations:** 1 OSCARE, Organisation for Burns, Scar After-care and Research, Van Roiestraat 18, B-2170 Antwerp, Belgium; 2 Department of Rehabilitation Sciences, KU Leuven, Tervuursevest 101, box 1500, 3001 Heverlee, Belgium; 3 Department of Rehabilitation Sciences and Physiotherapy, University of Antwerp, Universiteitsplein 1, 2610 Antwerp, Belgium; 4 Department of Clinical and Lifespan Psychology, Vrije Universiteit Brussel, Pleinlaan 2, 1050 Brussels, Belgium

**Keywords:** Vacuum massage, Endermology, Vacuotherapy, Depressomassage, Scars, Hypertrophic, Physical, Physiological, Intervention

## Abstract

Vacuum massage is a non-invasive mechanical massage technique performed with a mechanical device that lifts the skin by means of suction, creates a skin fold and mobilises that skin fold. In the late 1970s, this therapy was introduced to treat traumatic or burn scars.

Although vacuum massage was invented to treat burns and scars, one can find very little literature on the effects of this intervention. Therefore, the aim of this review is to present an overview of the available literature on the physical and physiological effects of vacuum massage on epidermal and dermal skin structures in order to find the underlying working mechanisms that could benefit the healing of burns and scars. The discussion contains translational analysis of the results and provides recommendations for future research on the topic.

An extended search for publications was performed using PubMed, Web of Science and Google Scholar. Two authors independently identified and checked each study against the inclusion criteria.

Nineteen articles were included in the qualitative synthesis. The two most reported physical effects of vacuum massage were improvement of the tissue hardness and the elasticity of the skin. Besides physical effects, a variety of physiological effects are reported in literature, for example, an increased number of fibroblasts and collagen fibres accompanied by an alteration of fibroblast phenotype and collagen orientation. Little information was found on the decrease of pain and itch due to vacuum massage.

Although vacuum massage initially had been developed for the treatment of burn scars, this literature review found little evidence for the efficacy of this treatment. Variations in duration, amplitude or frequency of the treatment have a substantial influence on collagen restructuring and reorientation, thus implying possible beneficial influences on the healing potential by mechanotransduction pathways. Vacuum massage may release the mechanical tension associated with scar retraction and thus induce apoptosis of myofibroblasts. Suggestions for future research include upscaling the study design, investigating the molecular pathways and dose dependency, comparing effects in different stages of repair, including evolutive parameters and the use of more objective assessment tools.

**Electronic supplementary material:**

The online version of this article (doi:10.1186/s41038-016-0053-9) contains supplementary material, which is available to authorized users.

## Background

**Fig. 1:**
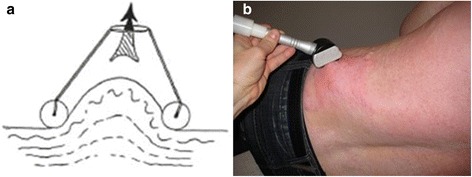
**a** Vacuum massage creates a skin fold and mobilises that skin fold. **b** The application of vacuum massage on a patient with burn scars

Vacuum massage is also known as depressomassage, vacuotherapy or Endermologie®. It is a non-invasive mechanical massage technique performed with a mechanical device that lifts the skin by means of suction, creates a skin fold and mobilises that skin fold [1–3] as displayed in Fig. 1a, b.

Vacuum massage originates from cupping therapy, a traditional Chinese medicine therapy dating back at least 2000 years [4]. A local suction on the skin is created using heat or mechanical forces. In the late 1970s, Louis-Paul Guitay developed the Endermologie® system, utilising a mechanical method to copy the manual massage techniques by means of negative pressure. This method allowed him to perform the massage in a more consistent and less time consuming way [5]. As from that moment on, Endermologie® or vacuum massage has been frequently used to treat traumatic or burn scars.

Although vacuum massage was invented to treat burns and scars [5], one can find very few literature on the effects of this intervention. Soon after its development the device is being used extensively in Europe to treat trauma and burn scars [2, 5]. During this use, care providers soon noticed its ability to improve the appearance of cellulite and consequently most studies mainly focused on lipodystrophy to investigate its working mechanism. The number of studies concerning cellulite is three times higher than this concerning burns or scars. Therefore, performing a review on the effects of depressomassage on the different skin layers could provide us with more information than a search for its effects on burns and scars alone.

The aim of this paper is to present an overview of the available literature with the physical and physiological effects of vacuum massage on epidermal and dermal skin structures. This was done in order to find the underlying working mechanisms of Endermologie® that could benefit the healing of burns and scars. Analysing the physical and physiological effects of this treatment can increase insights in the influence on the scarring process and may clarify the outcome. The discussion contains translational analysis of the accumulated results and provides recommendations for future research on the topic.

## Review

### Methods

#### Inclusion criteria

After establishing the research question, an extended search strategy was developed. Studies of the following categories were eligible to be included:

- Randomised controlled trial (RCT) or controlled clinical trial (CCT)
- Cohort studies, case control studies or cross-sectional studies
- Pilot studies
- Studies on animal models or human models
- Studies on the physical and physiological effects on epidermal, dermal and hypodermal skin structures
- Articles published between 1990 and 2016
- Articles written in English and French

All the eligible studies needed to examine the effects of vacuum massage. Studies on vacuum massage combined with other interventions were excluded. Animal studies were found eligible to the extent that the porcine model was used. This model is frequently used as a model for human cutaneous wound healing [6–8].

An extended search of publications was performed using PubMed, Web of Science and Google scholar. All searches started on January 1 1990 and ended on January 1 2016. The search terms included (*depressomassage OR “mechanical massage” OR endermologie OR “vacuum massage” OR suction massage) AND (burns OR scars OR fibroblast OR collagen OR elastin OR skin OR dermis OR epidermis OR “TGF beta” OR “connective tissue”).* Two authors independently identified and checked each study against the inclusion criteria.

#### Data extraction and quality assessment

The two authors extracted data from the included publications. The extracted data included authors and title of study, year of publication, patient population, study size and methodological information. Other extracted data included outcomes and adverse effects. The methodological quality of the included studies was assessed using the corresponding Scottish Intercollegiate Guidelines Network (SIGN) methodology checklists [9]. Next to this, a self-developed Literature Evaluation Scale for Scars (LESS-scale, see Additional file 1: Appendix A), adapted from the Miller Methodological Quality Rating Scale [10] and supplemented by elements from SIGN [9] and the International Conference on Harmonisation/Good Clinical Practice (ICH/GCP) guidelines [11], was used. The available scales in the literature lack important information for methodological evaluation of scar research like follow-up length, differentiating objective from subjective outcome or the use of the appropriate statistical analyses. We trust that this scale is a balanced and fair representation of the important factors to be detected in scar related literature (see Additional file 2: Appendix B).

### Study characteristics

**Fig. 2:**
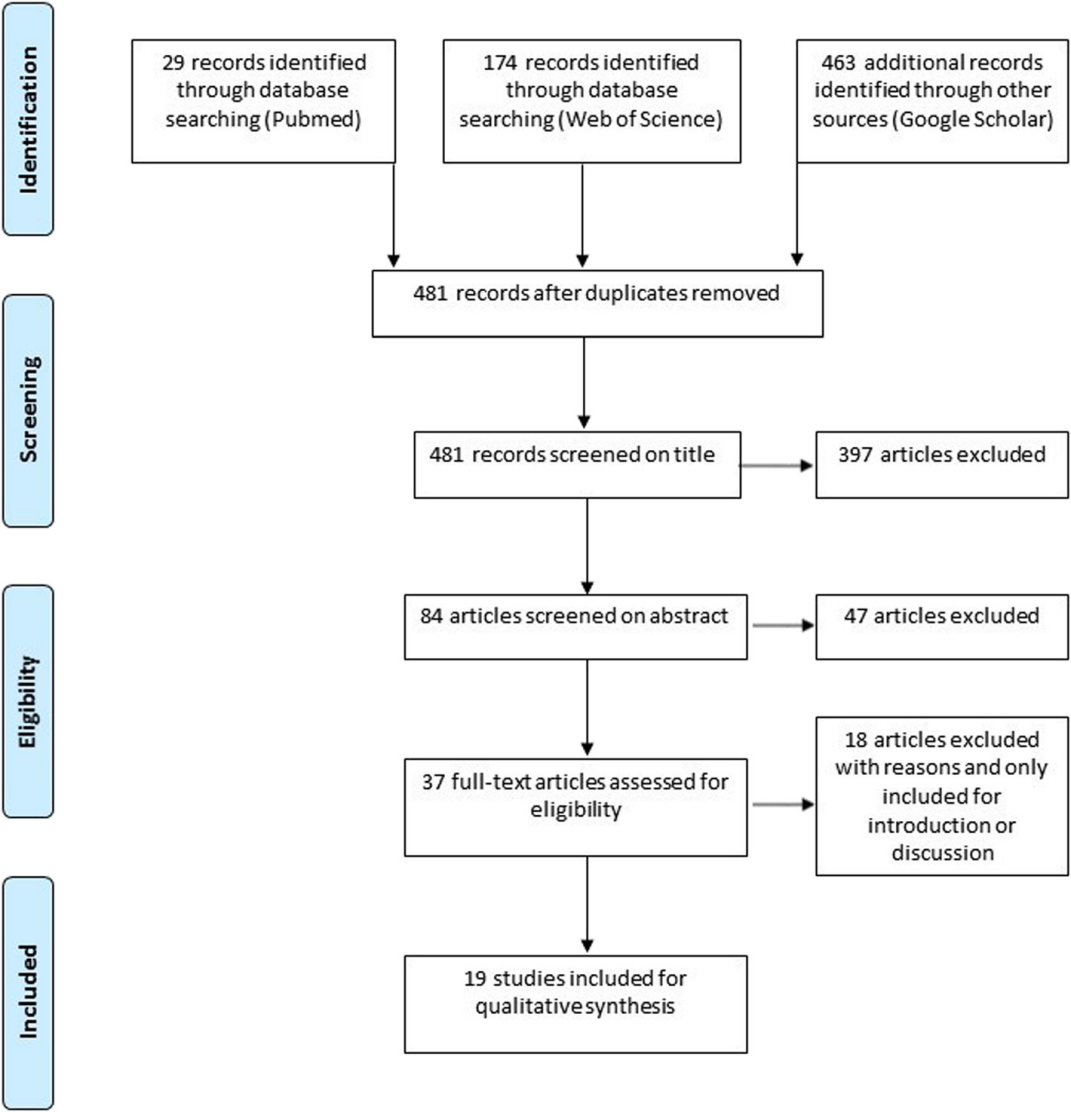
Flow diagram

The flow diagram of this review is shown in Fig. 2. An extended search of PubMed, Web of Science and Google Scholar identified 481 citations after removing the duplicates. After being screened on title and abstract 444 records were excluded. We assessed 37 full-text articles for eligibility, and finally, 19 full-text articles were included in the qualitative synthesis. The main reasons for exclusion were depressomassage combined with other interventions (*n* = 7), the targeted skin layer did not include the dermis or epidermis (*n* = 9) and the wrong study design/article format (white paper instead of a scientific article) (*n* = 2).

Among the included trials, eight of them were pilot studies with pre- or post- treatment evaluations [3, 12–18], four were controlled clinical trials [19–22] and seven were identified as randomised controlled trials [1, 23–28].

Ten trials investigated epidermal and dermal structures [3, 14, 17–21, 25, 27, 28], seven trials targeted the dermal/hypodermal junction [1, 13, 15, 16, 23, 24, 26] and in two studies it was unclear which skin layers were investigated [12, 22].

The total number of patients enrolled was 1002. Only two of the studies enrolled a significant number of patients (738 combined) [12, 22], unfortunately these studies lacked valid and reliable outcome measures (the vitro-pressure test and the cutaneous stretch test were at the time of publication not validated or tested for its reliability). Six trials [14, 17–19, 26, 27] studied the effects on 20 to 70 patients and 11 trials [1, 3, 13, 15, 16, 20, 21, 23–25, 28] were set up with less than 20 patients. This low sample size seemed to be common in interventional studies on burns and scars.

Two studies investigated the cutaneous and systemic effects of vacuum massage on animal models with similar metabolism and skin architecture as human beings [1, 23]. Lipodystrophy or cellulite was the target subject of four studies [13, 20, 21, 26]. Physical, physiological and metabolic effects of depressomassage on healthy skin were discussed in five studies [2, 14–16, 28]. One single study examined the effects on ageing skin [19]. In three studies, the investigated conditions were scar-like pathologies [3, 17, 24] (pathologies with characteristics that were similar to scar characteristics like stiffness or indurations) and eventually only four studies examined the effects of vacuum massage on scars [12, 18, 22, 27]. All these study characteristics are summarised in Table 1, ranked from a high to a low methodological score. The articles that scored high in the SIGN methodology checklist or the LESS scale distinguished themselves from others in the study design, baseline equality, the reliability and validity of the outcome measures and the use of appropriate statistics.

**Table 1:** Basic information on the papers included for qualitative analysis

Paper	Aetiology	Study design	Targeted skin layer	Patients (*n*)	Assessment	SIGN score	LESS score
Adcock et al. 2001 [23]	Pig skin	RCT	Hypodermis	4	Intra-dermal tonometry	++	17
Adcock et al. 1998 [1]	Pig skin	RCT	Dermis/hypodermis	12	Histology	++	16
Revuz et al. 2002 [19]	Ageing skin	CCT	Dermis	24	Subjective assessment of skin laxity and skin loosening, stereophogrammetry, cutometer	++	16
Moseley et al. 2007 [24]	Scar-like	RCT	Dermis/hypodermis	10	Likert scale, tonometry	++	16
Bourgeois et al. 2008 [27]	Scars	RCT	Epidermis/dermis	20	Subjective assessment of pain, itch, tightness, erythema and skin smoothening, profilometry	++	13
Lucassen et al. 1997 [15]	Healthy skin	Pre/post	Dermis/hypodermis	19	High-frequency ultrasound	+	13
Watson et al. 1999 [2]	Healthy skin	RCT	Epidermis/dermis	5	Laser-Doppler imaging, lymphoscintigraphy, venous flowmetry	+	13
Ortonne et al. 2003 [26]	Lipodystrophy	RCT	Dermis/hypodermis	30	High-frequency ultrasound, fringe projection, skin fold thickness	+	13
Innocenzi et al. 2003 [21]	Lipodystrophy	CCT	Epidermis/dermis	15	Quantitative histology	+	11
Monteux et al. 2008 [13]	Lipodystrophy	Pre/post	Dermis/hypodermis	9	Skin fold thickness	+	11
Marques et al. 2011 [16]	Healthy skin	Pre/post	Hypodermis	12	Gene profiling, micro-array	+	11
Innocenzi et al. 2002 [20]	Lipodystrophy	CCT	Epidermis/dermis	12	Descriptive histology	+	10
Scuderi et al. 2008 [28]	Healthy skin	RCT	Epidermis/dermis	10	Subjective assessment of skin smoothening and skin tone	0	10
Majani et al. 2013 [18]	Scars	Pre/post	Epidermis/dermis	26	Subjective assessment of skin smoothness, pain, tenderness, oedema and aesthetic improvement, histology	0	10
Marquez-Rebollo 2014 [17]	Scar-like	Pre/post	Epidermis/dermis	70	Number of indurations	0	10
Gavroy et al. 1996 [22]	Scars	CCT	Details not available	606	Test de glissement	0	7
Lattarulo et al. 2001 [14]	Healthy skin	Pre/post	Epidermis/dermis	34	Laser-Doppler imaging, tcpO2	0	7
Worret et al. 2004 [3]	Scar-like	Pre/post	Dermis	10	Subjective assessment of pain, colour and elasticity, Quality of Life, cutometer	0	7
Delprat et al. 1995 [12]	Scars	Pre/post	Details not available	132	Test de glissement	0	5

*RCT* randomised controlled trial, *CCT* controlled clinical trial, *tcpO2* transcutaneous oxygen pressure

### General, physical and physiological effects

The effects of depressomassage on dermal and epidermal skin layers may be divided into three main sub-groups: General effects, physical effects and physiological effects.

#### General effects

General effects are defined as the effects inherent to the intervention itself or to the individual who performs the treatment. In five studies, the measured effects were dependent on the number of treatments [1, 15, 19, 23, 26]. The more treatments, the higher the effect. Next to this ascertainment, Adcock et al. [23] also discovered that the principal force applied to the tissue during therapy depended on the particular type of manoeuver performed, with the suction and the roller tension being minor forces. Moreover, they observed a higher decrease of tension in thicker tissue.

In four studies, the results showed a setback after a follow-up period without treatment [15, 24, 26, 27], but one study demonstrated the opposite [19].

#### Physical effects

A summary of the different physical effects is set out in Table 2. An improvement of the tissue hardness and the elasticity of the skin were the two most observed effects [3, 17, 18, 23, 24, 27, 28]. However, most of these studies used subjective methods to quantify these effects. Other reported physical effects were decreased skin fold thickness [13, 26], decreased face volume [19], improved skin laxity [19, 28], increased epidermal thickness [20, 21], improved skin roughness [26, 27] and decreased redness [3, 27].

**Table 2:** Overview of the physical effects of vacuum massage on epidermis/dermis/hypodermis

Paper	Tissue hardness subjective	Tissue hardness objective	Elasticity subjective	Elasticity objective	Skin fold thickness	Scar adhesions	Face volume	Skin laxity	Epidermal thickness	Skin roughness	Colour subjective
Delprat et al. 1995 [12]						Decreased					
Gavroy et al. 1996 [22]						Decreased					
Adcock et al. 2001 [23]		Decreased									
Revuz et al. 2002 [19]				No changes			Decreased	Decreased			
Innocenzi et al. 2002 [20]									Increased		
Innocenzi et al. 2003 [21]									Increased		
Ortonne et al. 2003 [26]					Decreased					Decreased	
Worret et al. 2004 [3]	Decreased		Increased	Increased							Decreased
Moseley et al. 2007 [24]	Decreased	Decreased	Increased								
Bourgeois et al. 2008 [27]	Decreased									Decreased	Decreased
Monteux et al. 2008 [13]					Decreased						
Scuderi et al. 2008 [28]	Decreased							Decreased			
Marquez-Rebollo et al. 2014 [17]	Decreased		Increased								
Majani et al. 2013 [18]	Decreased		Increased								

#### Physiological effects

Table 3 shows a wide variety of physiological effects which separately only occurred in one or two studies at the most. Some of the investigations revealed very interesting effects, such as an increased number of fibroblasts and collagen fibres. These observations were accompanied by an alteration of fibroblast phenotype and collagen orientation [19, 20, 26]. Only two studies mentioned a decrease of pain and itching [3, 27].

**Table 3:** Overview of the physiological effects of vacuum massage on epidermis/dermis/hypodermis

Paper	Blood perfusion	Dermo-hypodermal junction	Collagen content	Collagen orientation	Venous flow velocity	Lymphatic flow	tcpO2	Fibroblasts	Fibroblast phenotype	Dermal interstitial space	Superficial vascular surface	Pain	Itch	Altered gene profile
Lucassen et al. 1997 [15]						Decreased								
Adcock et al. 1998 [1]						Decreased								
Watson et al. 1999 [2]		Decreased												
Lattarulo et al. 2001 [14]				No changes			Decreased	Decreased						
Revuz et al. 2002 [19]									Increased					
Innocenzi et al. 2002 [20]									Increased					
Innocenzi et al. 2003 [21]					Decreased					Decreased				
Ortonne et al. 2003 [26]	Decreased		Increased	Increased							Decreased			
Worret et al. 2004 [3]	Decreased	Decreased	Increased									Decreased		
Bourgeois et al. 2008 [27]	Decreased									Decreased	Decreased	Decreased	Decreased	
Marques et al. 2011 [16]					Decreased									Yes
Majani et al. 2013 [18]	Decreased							Decreased						

*tcpO2* transcutaneous oxygen pressure

### Treatment parameters

A very remarkable finding is that 17 out of 19 investigations were conducted with an LPG® Endermologie® device (LPG Systems, Valence, France), which increases the risk of bias. In only six studies, the applied intensity setting was mentioned and only one of them used a universal intensity indication. Therefore, it was difficult to compare different trials [1, 2, 15, 22, 23, 28]. The treatment time was indicated in 18 of the 19 studies and took on average 18 min. The treatment frequency varied significantly between one and seven treatments per week. This also applied to the total number of treatments ranging from 1 to 40. In the majority of the studies, the applied techniques were well-described or the authors referred to a pre-defined protocol which was however not described in the paper. Table 4 summarises all this information.

**Table 4:** Overview of treatment parameters

Paper	Device	Intensity	Techniques	Treatment time in minutes	No. of treatments	Frequency/week
Delprat et al. 1995 [12]	LPG®	Details not available	Details not available	5	Min. 15	Between 3 and 7
Gavroy et al. 1996 [22]	LPG®	5	Technique selection based on pathology	4	Details not available	Details not available
Lucassen et al. 1997 [15]	Details not available	200 mBar	Unidirectional pinch/roll pulsating	15	40	3
Adcock et al. 1998 [1]	LPG®	Increasing	Smoothing, bouncing, figure eight, popping	10	4, 10 or 20	1 or 2
Watson et al. 1999 [2]	LPG®	6 or 7	Smoothing, bouncing, figure eight	20	2	2×/day
Adcock et al. 2001 [23]	LPG®	3–5–7–9	Smoothing, bouncing, figure eight, popping, kneading	10	4, 10 or 20	1 or 2
Lattarulo et al. 2001 [14]	LPG®	Details not available	Details not available	20	1	1x/day
Revuz et al. 2002 [19]	LPG® Lift 6	Details not available	Details not available	15	24	3
Innocenzi et al. 2002 [20]	LPG®	Details not available	Details not available	11	14	2 or 3
Innocenzi et al. 2003 [21]	LPG®	Details not available	Details not available	11	14	2 or 3
Ortonne et al. 2003 [26]	LPG®	Details not available	Pre-defined in practical protocol^a^	35	16, 22 or 28	2
Worret et al. 2004 [3]	LPG®	Details not available	Pre-defined “scars” programme of device	Details not available	13	1
Moseley et al. 2007 [24]	LPG®	Details not available	Pre-defined in practical protocol^a^	30	16	4
Bourgeois et al. 2008 [27]	LPG®	Details not available	Pre-defined in practical protocol^a^	10	15	3
Monteux et al. 2008 [13]	LPG®	Details not available	Pre-defined in practical protocol^a^	Details not available	12	3
Scuderi et al. 2008 [28]	ICOONE®/LPG®	10/5	Pre-defined in practical protocol^a^	35	10	2
Marques et al. 2011 [16]	LPG®	Details not available	Pre-defined in practical protocol^a^	30	12	2
Majani et al. 2013 [18]	LPG®	Details not available	Frequency: continuous for mature scars, 4 Hz for edema, 8-16 Hz for vascularization	Details not available	Between 8 and 20	2
Marquez-Rebollo et al. 2014 [17]	LPG®	Details not available	Forward, backward, sidelong and diagonal movements	15–40	12	2

^a^The techniques were cited by the authors as pre-defined in a practical protocol which was not described in the article

### Bias

A key part of a review is to consider the risk of bias in the results of each of the eligible studies.

To avoid selection bias, no systematic differences between baseline characteristics of the compared groups were allowed. Of the 19 included studies only 4 met this criterion [1, 19, 23, 27]. The risk for performance bias and detection bias was considered to be very high since we did not find a report on outcome assessment blinding and in only two studies [19, 24] the participants were blinded. This could be explained by the nature of this treatment, which impeded a placebo treatment (vacuum suction of the skin could not be imitated without causing the same physical and physiological effects). Ten out of 19 studies reported dropouts [1, 2, 13, 15, 17–21, 23], therefore avoiding attrition bias. Five studies [12, 14, 21, 22, 28] were not published in a peer reviewed journal and only four studies [1, 16, 23, 24] cited any information about conflict of interest, thus created a possible reporting bias.

### Animal experiments

#### Mechanotransduction

Adcock et al. [1, 23] used Yucatan mini-pigs and Yorkshire cross pigs in their trial. Their anatomical and physiological skin characteristics resemble most to humans who develop normotrophic or slight hypertrophic scars. Therefore, we could only transfer their findings towards humans with a low or intermediate level of scarring. Dense longitudinal collagen bands were prominently displayed, especially in mid to deep sub-dermal tissues. Colorimetric analysis of subcutaneous tissues revealed significant increases in the percentage of tissue collagen in the treated tissues from both intermediate-term and long-term treatments. The collagen content of the subcutaneous tissues increased on average from 27 % to as high as 130 % in the long-term subjects. The thickness of the tissue correlated highly with the positive effects of the treatment. The changes in sub-dermal tissue architecture resulting from Endermologie® treatment were proportional to the number of treatments performed. Additionally the study indicated that the force applied to the tissue depended on the manipulation of the treatment head, with the suction and the roller tension being of minor interest. This could indicate that the type of mechanical force (shear, strain or compression) is of higher importance for the extra cellular matrix (ECM) remodelling effect than the intensity of the force.

As frequently mentioned in literature, external mechanical stimuli could lead to alterations in collagen deposition and orientation [29–34], also known as mechanotransduction. Recent studies showed how mechanotherapies targeted particular cells, molecules, and tissues [35, 36]. The role of mechanical forces in negative pressure wound therapy and shockwave therapy were the topic in many studies [37, 38]. The dose dependency of collagen remodelling by mechanical forces was already emphasised in prior studies [39–42]; so the findings described by Adcock et al. [1, 23] were to be expected and mechanotransduction would probably be the working mechanism of vacuum massage. Yet, the question remains whether these results also apply to hypertrophic scars?

#### Animal wound-healing models

Our choice to include studies performed on a porcine model in this review was motivated by the similarity of skin characteristics between man and pig [6]. The medical literature described several in vitro and in vivo wound-healing models. The choice for an animal model depended on a number of factors, including availability, cost, ease of handling and anatomical similarity to humans. Small mammals were frequently used for wound-healing studies, nevertheless, these mammals differ from humans in a number of anatomical and physiological ways. Anatomically and physiologically, pig skin resembles to human skin. The many similarities between man and pig make the porcine model an excellent tool for the evaluation of therapeutic agents destined for use in human wounds [6]. Most recently, the female red Duroc pigs were validated as a new model to study hypertrophic scarring, demonstrating its similarity to human scars in different ways. Molecular expression patterns indicated that the healing phenotype of female red Duroc pigs correlated very well with those of human hypertrophic scarring models [7]. The female Yorkshire pig had been demonstrated to heal in a very different manner, more resembling human normotrophic scarring [8]. As a consequence, we must be careful to transfer results of studies on healthy animal skin to hypotheses concerning treatment efficacy of therapies for hypertrophic scarring in humans. Specifically, it was unknown whether the same factors that initiated hypertrophic scarring in these species were involved in human diseases.

#### Suggestions

Future studies examining the effects of vacuum massage on the scarred animal skin should make use of the female red Duroc pig. It would also be interesting to investigate and to compare the molecular pathways involved in hypertrophic scarring for red Duroc pig and man. Another recommendation would be to include the dose dependency in future investigations of the effects of depressomassage by implementing sub-groups differentiating treatment intensity, frequency and duration.

### Lipodystrophy

Numerous studies have investigated the effects of vacuum massage on lipodystrophy or cellulite. We only included four of them since they were the only ones to reveal any relevant information for this review by investigating effects on epidermal, dermal or hypodermal skin layers [13, 20, 21, 26].

Innocenzi et al. [20, 21] performed descriptive and quantitative histology after 6 weeks of Endermologie® treatment. The results showed an increased number of fibroblasts compared to the control side, a thickened epidermis in the majority of cases (although not significant) and a decrease of dermal interstitial space, which was also confirmed by Ortonne et al. [26]. The fibroblasts changed into a “secreting” phenotype at the treated side, when their phenotype remained “normal” at the control side [20]. A significant improvement in skin fold thickness which lasted for 50 % after 6 months of follow-up, regardless of follow-up treatment or not, was observed by two authors [13, 26].

The question remained whether it is permissible to interpret these results into hypotheses concerning the effects of vacuum massage on scarred skin.

Several theories of cellulite pathophysiology have been formulated over the past years. These mechanisms were reflected in the different names given to describe cellulite, e.g., nodule liposclerosis, gynoid lipodystrophy, etc. In general, these theories can be separated into three groups:

- Increase of the water content, leading to oedema [43].
- A change in the local microcirculation [44], with the progressive development of lymphedema.
- An abnormal arrangement of collagen structure in the tissue and continuous ECM remodelling [45, 46].

We were particularly interested in the last group since these features were closely linked to the process of skin scarring. New insights in the pathophysiology of lipodystrophy considered the excessive production of low molecular weight hyaluronan fragments in hypertrophic fat tissue to play an important role in cellulite development [47]. These fragments induced different types of adipose tissue fibrosis of which fibrillar fibrosis consisted mainly of collagen types I and III. The collagen fibres were organised in thick bundles and created tension in the surrounding fat tissue. The tension was dependent on the thickness of the bundles and influenced the orientation of the collagen fibres. This type of fibrosis looked very similar to dermal scarring [47].

Furthermore, adipocytes seemed to play an important role in epidermal homeostasis during hair follicle regeneration and wound healing [48, 49], hereby emphasising the constant interaction of components presented in all skin layers.

All these findings suggested that outcomes of clinical trials which investigated fibrillar adipose tissue fibrosis could be transferred to dermal scarring, but since none of the included studies clearly defined the diagnosis of lipodystrophy, caution should be used when interpreting these results.

To date, the pathophysiology of cellulite and skin scarring has been investigated independently of each other. The above mentioned findings indicated that their mechanisms of action were closer related than assumed. Future studies in the molecular field should target all skin layers to explore the signalling pathways involved with tissue fibrosis in general.

### Apoptosis

Almost 85 years ago, Howes et al. described the three classic phases of wound healing [50]—inflammation, proliferation and maturation. The end result of a normal wound healing was a flat scar, with little fibrosis and minimal if any wound contraction. Prolonged inflammation, excessive collagen deposition as a result of continuing fibroblast proliferation and delayed apoptosis of myofibroblasts led to hypertrophic, retracted scars [51–53]. All treatments for scarring should therefore be directed at reducing these signs.

Marques et al. [16] investigated the impact of mechanical massage on gene expression profile. An interesting discovery was that anti-inflammatory gene expression seemed to be up-regulated (MMPs, IL-10, IL-1RN, CD163). Although this could account for some defence response of the adipose tissue to massage in this study, future research could provide us with more information about these anti-inflammatory effects.

The suction forces generated by vacuum massage could elicit an array of mechanical forces within the tissues, associated with a relaxation of those mechanical forces [52, 54]. Once stress forces on a wound were relieved, apoptosis of myofibroblasts would occur [54]. This finding implies that depressomassage may release the mechanical stress associated with scar retraction and thus induce apoptosis. This can be another plausible theory for its mechanism of action to improve the outcome of (burn) scars.

### Stage of repair

The reported increase of epidermal thickness and collagen content seems contradictory with the reduction of hypertrophic scarring. It is very similar to the characteristics of hypertrophic scars in the proliferative phase. However, these findings are in line with previously reported data from recent clinical trials which strongly support the use of laser- and light-based therapies for the treatment of hypertrophic burn scars [55–57]. This throws up many questions in need of further investigation. From the seven studies that investigated scars or scar-like pathologies, four of them [3, 17, 24, 27] only included patients with scars that were in their late maturation phase and for three of them [12, 18, 22] scar age (time after full wound closure) was neither an inclusion or exclusion criterion. The authors of the three previous articles did report different treatment guidelines for young and old scars, thus assuming that the stage of repair could be an important parameter for treatment approach, a hypothesis which was acknowledged by several authors [58–60]. In this context, it would be advisable to compare the effects of vacuum massage in different stages of repair or to include evolutional parameters such as colorimetric assessment or skin barrier function [61, 62] to investigate whether healing could be accelerated by this treatment.

## Conclusion

Although vacuum massage initially had been developed for the treatment of burn scars, this literature review found little evidence for the efficacy of this treatment. Furthermore the overall quality of these studies turned out to be poor, thus providing very little relevant information. The heterogeneous population and the wide diversity of study designs make it very hard to translate the previously mentioned results towards the burns and scars population in humans. Although the present study confirms previous findings of vacuum massage on dermal and epidermal skin structures and contributes additional evidence for the working mechanism of vacuum massage as an anti-scarring agent, the results should be confirmed by studies on human models. Variations in duration, amplitude or frequency of the treatment have a substantial influence on collagen restructuring and reorientation, thus implying possible beneficial influences on the healing potential by mechanotransduction pathways. Vacuum massage may release the mechanical tension associated with scar retraction and thus induce apoptosis of myofibroblasts. Suggestions for future research include upscaling the study design, investigating molecular pathways and dose dependency, comparing effects in different stages of repair, including evolutional parameters and the use of more objective assessment tools.

## Abbreviations

CCT, controlled clinical trial; CD163, cluster of differentiation 163; ECM, extra-cellular matrix; IL-10, interleukin-10; IL-1RN, interleukin-1 receptor antagonist; LESS, Literature Evaluation Scale for Scars; MMP, matrix metalloproteinases; RCT, randomised controlled trial

## Additional files

Additional file 1: Appendix A. LESS-scale: Literature Evaluation System for Scars (Adapted from Miller Methodological Rating Scale 1995). (DOCX 12 kb) Additional file 2: Appendix B. Details of scoring for the LESS-scale. (XLSX 9 kb)
