# Supplementary material for: The physical and physiological effects of vacuum massage on the different skin layers: a current status of the literature
Source: Burns Trauma. 2016 Sep 19;4:34. doi: 10.1186/s41038-016-0053-9 (PMC5027633; doi:10.1186/s41038-016-0053-9)
Supplement: Additional file 1: Appendix A. — LESS-scale: Literature Evaluation System for Scars (Adapted from Miller Methodological Rating Scale 1995). (DOCX 12 kb) [file 41038_2016_53_MOESM1_ESM.docx]

Appendix A:

LESS-scale: Literature Evaluation System for Scars (Adapted from Miller Methodological Rating Scale 1995)

Methodological Attributes Points Awarded

Research Question

0. No clear and well defined research question addressed

1. The study addresses a clear and well defined research question

Study Design

1. Single group pretest-posttest

2. Quasi-experimental (nonequivalent control group / non-randomization)

3. Randomization with control group

Replicability

0. Intervention or follow-up description insufficiently detailed

1. Procedures contain sufficient detail

Baseline

0. No baseline scores, client characteristics or measures reported

1. Baseline scores, client characteristics or measures reported

2. Baseline equality

Quality control

0. No intervention standardized specified

1. Intervention standardized by manual, procedures, specific training and so forth

Follow-up length

0. Less than 6 months

1. 6-11 months

2. 12 months or longer

Follow-up rate

0. Less than 80% completion or no report

1. 80% to 100% completion

Compliance

0. No report on compliance

1. Compliance rate as exclusion criterion

Objective verification

0. No objective verification of records

1. Verification of records (paper records, medical charts, diagnostic interviews, data storage)

Dropouts

0. No discussion or enumeration of dropouts or dropout excluded from analysis

1. Intervention dropout enumerated

Independent

0. Non-blinded

1. Single-blinded

2. Double-blinded

Reliability of outcome measures (OM)

0. No reliable OM

1. Reliable subjective OM alone

2. Reliable objective OM alone

3. Reliable subjective and objective OM

Analyses

0. No statistical analyses conducted or clearly inappropriate analyses

1. Appropriate statistical analyses

Conflict of interest

0. No report of conflict of interest or industry sponsored

1. No conflict of interest reported

Review

0. Not published in a peer-reviewed journal

1. Published in a peer-reviewed journal

NOTE:

Scores could range from 0 (low) to 22 (high).

Adapted from Miller et al. (1995) and Vaughn and Howard (2003).
